# Exosomal miR-552-5p Regulates the Role of NK Cells in EMT of Gastric Cancer via the PD-1/PD-L1 Axis

**DOI:** 10.7150/jca.102360

**Published:** 2025-01-01

**Authors:** Jingwen Qin, Jinhua Yang, Haopeng Cui, Chunling Feng, Aiqun Liu

**Affiliations:** Department of Gastroenterology and Respiratory Internal Medicine & Endoscopy Center, Guangxi Medical University Cancer Hospital, Nanning, Guangxi 530021, P.R. China.

**Keywords:** gastric cancer, natural killer cells, exosomes, miR-552-5p, epithelial-mesenchymal transition

## Abstract

**Background:** While previous studies have established the role of exosomal miR-552-5p in promoting gastric cancer (GC) progression, the exact mechanisms through which it modulates the PD-1/PD-L1 axis to affect NK cell function and subsequently influence GC epithelial-mesenchymal transition (EMT) remain to be elucidated.

**Methods:** Western blot, transmission electron microscopy (TEM), and nanoparticle tracking analysis were used to characterize exosomes that were isolated from GC cell supernatants. Subcutaneous AGS cell injections expressing either Lv-miR-552-5p or Lv-NC were administered to nude BALB/C mice. Mice received intraperitoneal injections of anti-PD-L1 antibody (12.5 mg/kg) or isotype control IgG weekly for two weeks. Flow cytometry assessed NK cell proportions and activation receptor (NKG2D, NKp46) and PD-L1 expression. ELISA measured cytokine levels (IFN-γ, granzyme B, perforin). Immunohistochemistry evaluated EMT marker expression in tumor tissues. An *in vitro* co-culture of NK cells with Exo-Lv-miR-552-5p or Exo-Lv-NC and GC cells was established. EMT protein expression in GC cells was analyzed by Western blot and immunofluorescence. Transwell assays and a tail vein-lung metastasis model in nude mice tested GC cell migration and invasion.

**Results:** Expression of NKG2D, NKp46, and PD-L1 was significantly reduced in Exo-Lv-miR-552-5p mice peripheral blood NK cell percentage. Increased treatment with PD-L1 inhibitors reversed the considerable reduction in IFN-γ, granzyme B, and perforin cytokine expression levels. Exosomal miR-552-5p overexpression in NK cells reduced E-cadherin expression while increasing N-cadherin and vimentin expression in GC cells, promoting migratory and invasive properties.

**Conclusions:** GC-derived exosomal miR-552-5p promotes EMT in GC by inhibiting NK cell activity via the PD-1/PD-L1 axis, which provides new insights into the role of exosomal miR-552-5p in GC progression and immune escape.

## Introduction

Gastric cancer (GC) occupies a prominent role in the global oncological landscape. The 2022 "Global Cancer Statistics Report" indicates that gastric cancer's incidence and mortality rates are the fifth highest among all malignancies globally 1. Despite the considerable progress in multidisciplinary therapy for GC, the enhancement in the 5-year postoperative survival rate remains modest, primarily attributed to the scarcity of sensitive prognostic biomarkers and efficacious therapeutic targets 2,3. Thus, a profound investigation into the molecular underpinnings of GC and the quest for innovative therapeutic approaches are of paramount importance.

Tumor progression is influenced not only by the neoplastic cells themselves but also by their microenvironment, particularly the host's immune response 4. As essential elements of the immune surveillance system, natural killer (NK) cells play a critical role in maintaining immunological homeostasis and inhibiting the formation of tumors 5-7. However, a diminution in NK cell efficacy has been observed within gastric cancer tissues and peripheral blood 8, and is intricately linked to tumor advancement and patient prognosis 9,10. Thus, clarifying the role of NK cells in GC could lead to the development of new treatment approaches.

Exosomes are minuscule extracellular vesicles, measuring 30-150nm in diameter, are laden with proteins, mRNA, miRNA, and other molecules that facilitate intercellular communication 11. Through a variety of ways, they can promote the growth of tumors, and the miRNA they contain is essential for regulating the actions of tumor cells and their reactions to immunotherapy 12. Our previous research has revealed that Exosomal miR-552-5p produced from gastric cancer cells can accelerate the growth and epithelial-mesenchymal transition (EMT) of gastric cancer 13. Furthermore, we discovered that Exosomal miR-552-5p affects the PD-1/PD-L1 axis, resulting in the reduction of NK cell activity 14.

Recent research has clarified how suppressive immune cells induce EMT 15, and a positive correlation has been established between EMT and the immunosuppressive state of these cells 16-18. Based on these findings, we propose that exosomal miR-552-5p may produce an immunosuppressive phenotype in NK cells, thus facilitating the EMT process in gastric cancer cells. Using both *in vivo* and *in vitro* experimental paradigms, the current investigation aims to validate this theory and elucidate the function and mechanism of exosomal miR-552-5p in modifying NK cells during gastric cancer EMT through the PD-1/PD-L1 axis.

## Materials and Methods

### Cell culture

The Chinese Academy of Sciences' ATCC cell bank provided the stomach cancer cell lines SGC-7901 and AGS. These were cultured in either Dulbecco's Modified Eagle's media (DMEM; Gibco) or RPMI 1640 media (Gibco, USA), both of which were enhanced with 1% penicillin-streptomycin and 10% fetal bovine serum (FBS; Biological Industries, Israel). The NK-92 cell line (Procell Life Sciences Ltd) derived from NK cells of patients with malignant non-Hodgkin's lymphoma, was maintained in NK cell-specific medium (PeproTech) or RPMI-1640 (Gibco) with 20% FBS and 100 IU/mL IL-2 (PeproTech, China). Every culture was kept in an incubator with 5% CO2 at 37 °C.

### Lentiviral transfection

Lentiviral plasmids for the overexpression of miR-552-5p and their negative controls were synthesized by Genechem (Shanghai, China). Cells were seeded in 6-well plates (5 × 10^4^ cells/ mL) and transfected with the lentiviral plasmids. After a 3-day culture, selection was performed using 1.5 μg/mL puromycin. Quantitative PCR confirmed the expression levels of miR-552-5p. The lentiviral plasmids were transfected into cells that had been plated in 6-well plates (5 × 10^4^ cells/ mL). Using 1.5 μg/mL puromycin, selection was carried out following a 3-day culture. The levels of miR-552-5p expression were verified by quantitative PCR.

### Exosome extraction

FBS devoid of exosomes was prepared by centrifugation at 100,000×g for 16 hours at 4 °C. SGC-7901/AGS cells were cultured in DMEM/ RPMI 1640 until reaching approximately 80% confluence, after which the medium was replaced with exosome-free conditioned medium. Following a 48-hour incubation, the culture medium was centrifuged at 300×g for 10 minutes and 2000×g for 20 minutes at 4 °C to remove cellular debris. Larger vesicles were extracted using a 30-minute 10,000×g centrifugation process. Following a 0.22 μm filter (Millipore, USA) filtering of the supernatant, exosomes were pelleted by centrifugation at 100,000×g for 70 minutes at 4 °C. After being resuspended in PBS, the pellet was centrifuged once more for 70 minutes at 100,000×g. The last exosome pellet was kept at -80 °C after being resuspended in PBS.

### Transmission Electron Microscopy (TEM)

Formvar carbon-coated copper grids were treated with exosomes, which were then left to adsorb for five minutes before being negatively stained with 2% uranyl acetate solution. The exosomes were inspected under an electron microscope (Hitachi, Japan) to determine their size and shape.

### Co-culture system

NK-92 cells were co-cultured with Exo-Lv-miR-552-5p or Exo-Lv-NC in the culture medium for 24 hours. A Transwell co-culture system with a 0.4μm pore size (Corning, USA) was used, with NK-92 cells in the upper chamber and gastric cancer cells (SGC-7901/AGS) in the lower chamber. The gastric cancer cells were taken for additional research after 48 hours.

### Transwell assay

To facilitate cell migration, 200 μL of serum-free media containing 1 × 10^5^ cells/mL was introduced to the upper chamber containing the resuspended gastric cancer cells. On Matrigel-coated membranes (Corning), cells were sown at a density of 2 × 10^5^ cells/mL for invasion. A medium containing 10% FBS was added to 500 μL of the lower chamber. Non-migrating cells were extracted after 48 hours, and the membrane was then fixed and stained with crystal violet. The number of migrated cells was measured under a microscope.

### Western blotting

Protein concentration was assessed using a BCA kit after cells or exosomes were lysed in radioimmunoprecipitation lysis buffer (RIPA) containing PMSF (100:1). Proteins were separated using a 10% SDS-containing polyacrylamide gel (SDS-PAGE) and subsequently placed onto PVDF membranes measuring 0.22-μm in diameter. After that, the membranes were blocked for 1.5 hours in TBST with 5% skim milk. PVDF membranes were treated with antibodies against the following proteins for an overnight period at 4 °C following three PBS washes: TSG101(Servicebio, China), CD63(Servicebio), ALIX (Servicebio), E-cadherin (CST, USA), N-cadherin (CST), vimentin (CST), or GAPDH (CST). After three PBS washes, the membrane is left to incubate for one hour at room temperature with a secondary antibody conjugated with HRP. Enhanced chemiluminescence was used to observe the protein bands, and GAPDH was used as a reference to normalize expression.

### Immunofluorescence (IF)

Gastric cancer cells were seeded onto cover glass slides in 12-well plates at a density of 2×10^5^ cells per slide and incubated for 24 hours to allow complete adhesion. After removing the 12-well plate, wash three times with PBS, then fix the cells with 4% paraformaldehyde (Thermo Fisher, USA) for 20 minutes. Subsequently, PBS containing 0.1% Triton X-100 was used for cell permeability for 15 min, and then cells were washed with PBS. Next, add the primary antibody (E-cadherin (Biyotime, China), N-cadherin (Biyotime), vimentin (Biyotime),) and incubate overnight at 4°C. After washing, the cells were incubated with fluorescently labeled secondary antibodies for 1 hour in the dark. Nuclei were stained with DAPI (Burlingame, CA) at room temperature in the dark for 5 minutes, and mount the slides. Finally, observe and photograph the experimental results under a fluorescence microscope (Olympus, Japan).

### Animal studies

Female BALB/c nude mice, aged four weeks, were procured from Guangxi Medical University's Animal Experimental Center and kept in SPF environments. The animal ethics committee of Guangxi Medical University approved all procedures used in animal experiments. AGS cells transfected with either LV-NC or Lv-miR-552-5p were injected subcutaneously into mice, which were divided into four groups at random (n = 5 per group). Tumor growth was monitored, and when tumors reached approximately 100 mm^3^, the Exo-Lv-miR-552-5p group received intraperitoneal injections of anti-PD-L1 antibody (Bio Xcell, USA) or isotype control IgG (Bio Xcell) weekly for two weeks a dose of 12.5 mg/kg. After 18 days, the xenografts were excised, frozen in liquid nitrogen, or preserved in 4% paraformaldehyde for further examination. Blood was also taken, and the mice were put to death by cervical dislocation. The formula used to compute the tumor volume was V = (width^2^ × length) / 2.

Four-week-old female BALB/c nude mice had their tail veins injected with reconstituted cells at a density of 2×10^6^ cells/mL in PBS. The Exo-Lv-miR-552-5p group received intraperitoneal injections of anti-PD-L1 antibody or isotype control IgG (Exo-Lv-miR-552-5p+aPD-L1 group or Exo-Lv-miR-552-5p+IgG group). Mice were slaughtered by cervical dislocation after 8 weeks, and H&E staining was used to determine the extent of metastases in the lung tissues.

### Antibodies and flow cytometry

Antibodies for flow cytometry included anti-mouse CD3-eFluor™ 450 (Thermo Fisher), anti- mouse CD314 (NKG2D) -PE (Thermo Fisher), anti- mouse CD335 (NKp46)-APC (Thermo Fisher) anti- mouse-CD49b-FITC (Thermo Fisher), anti-mouse CD274 (PD-L1)- (Thermo Fisher). Following the manufacturer's directions, cells were stained, and a flow cytometer (Beckman Coulter, USA) was used to examine the results. CytoFLEX software was used to process the data.

### ELISA

Collect peripheral blood from nude mice using EDTA anticoagulant tubes, centrifuge (4°C, 1000g, 20minutes), collect the supernatant to obtain plasma samples. Use ELISA kits (Meimian, China) to detect IFN-γ, GZMS-B, and perforin in the plasma samples of each group. Follow the specific instructions in the kit for the operation.

### Immunohistochemistry (IHC) and H&E staining

Each group's tumor xenografts were embedded in paraffin, cut, and fixed with 4% paraformaldehyde. The sections underwent two hours of incubation at 60 °C, followed by xylene dewaxed and an ethanol gradient dehydration. Following antigen repair, the sections were incubated for an additional night at 4°C with the following antibodies: anti-PD-L1 antibody (Proteintech, China), anti-E-cadherin (Servicebio), anti-N-cadherin (Servicebio), and normal non-immune animal serum. After 30 minutes of rewarming, sections were incubated for 10 minutes at room temperature with a secondary antibody. Ultimately, the slides were examined using light microscopy (Olympus BX43, Japan) after being stained with newly made 3,3′-diaminobenzidine (DAB) and hematoxylin.

The lung metastasis sections underwent a series of treatments, including xylene and an ethanol gradient, followed by immersion in hematoxylin solution, differentiation with hydrochloric alcohol, and eosin staining.

### Statistical analysis

GraphPad Prism version 9.5.0 for Windows was used to conduct statistical analysis. The results were displayed as mean ± SD. The statistical significance between two groups and among several groups was investigated using the independent sample t test and one-way analysis of variance (ANOVA). Results were deemed statistically significant when p<0.05.

## Results

### Isolation and identification of exosomes

**Figure 1:**
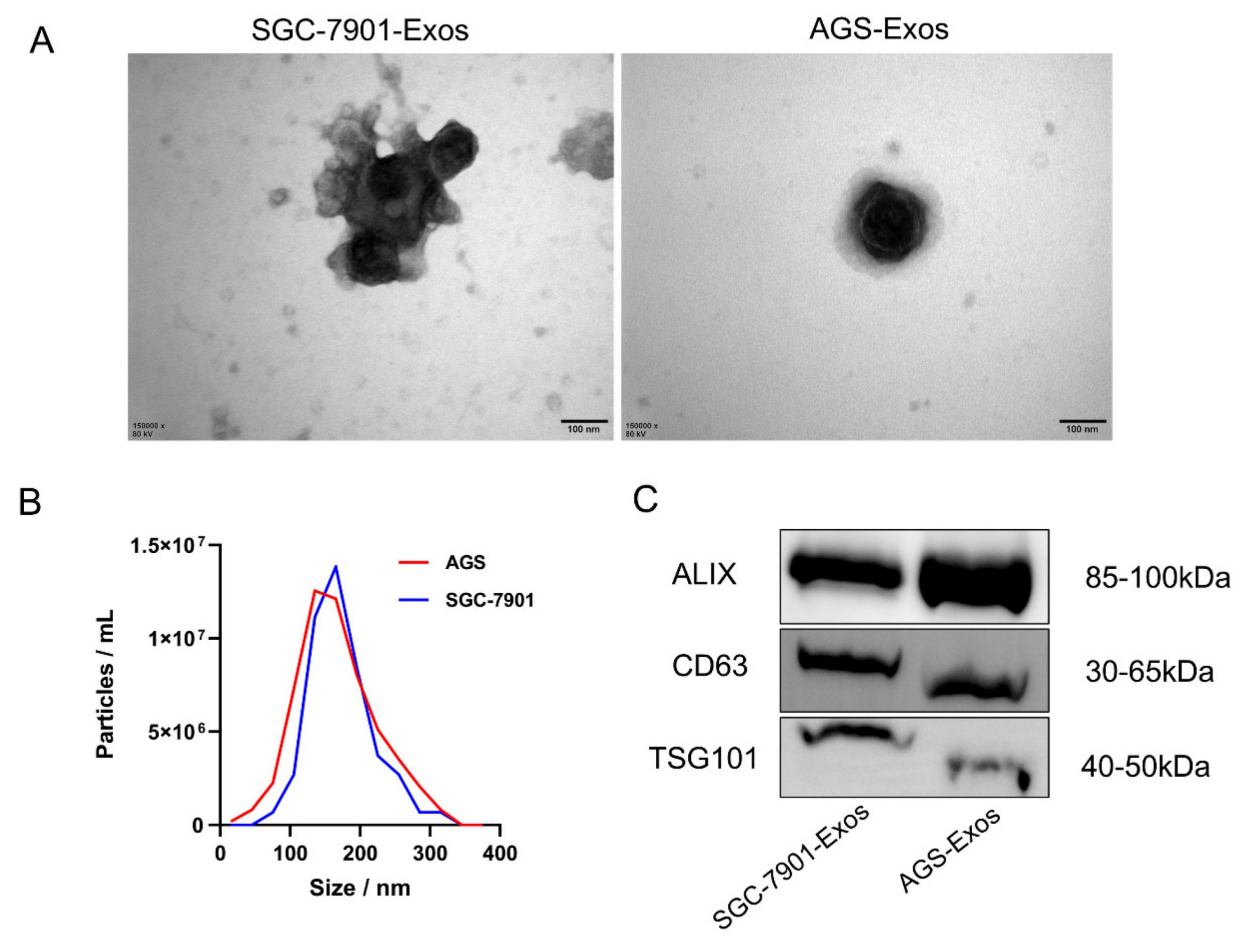
Identification of Exosomes. (A)TEM observation of the electron micrograph morphology of exosomes in the culture supernatant of SGC-7901 and AGS cells (scale bar, 100 nm); (B) NTA analysis of the diameter and density of exosomes; (C) Western blotting detection of the expression levels of exosomal marker proteins.

We used a gradient centrifugation technique to separate the exosomes from the exosome-depleted media in which we cultivated SGC-7901 and AGS cells. Subsequently, we validated the exosomes from three dimensions: morphology, size distribution, and expression of surface markers, using TEM, NTA, and Western blotting, respectively. The exosomes have a roughly spherical vesicular form, as seen by the TEM findings (Figure 1A). Exosome diameter was shown to be centered at 135 nm (Figure 1B) according to NTA analysis. Exosome markers (including CD63, TSG101, and ALIX) were verified to be expressed (Figure 1C) by Western blotting.

### Exosomal miR-552-5p induces NK cell dysfunction *in vivo*

**Figure 2:**
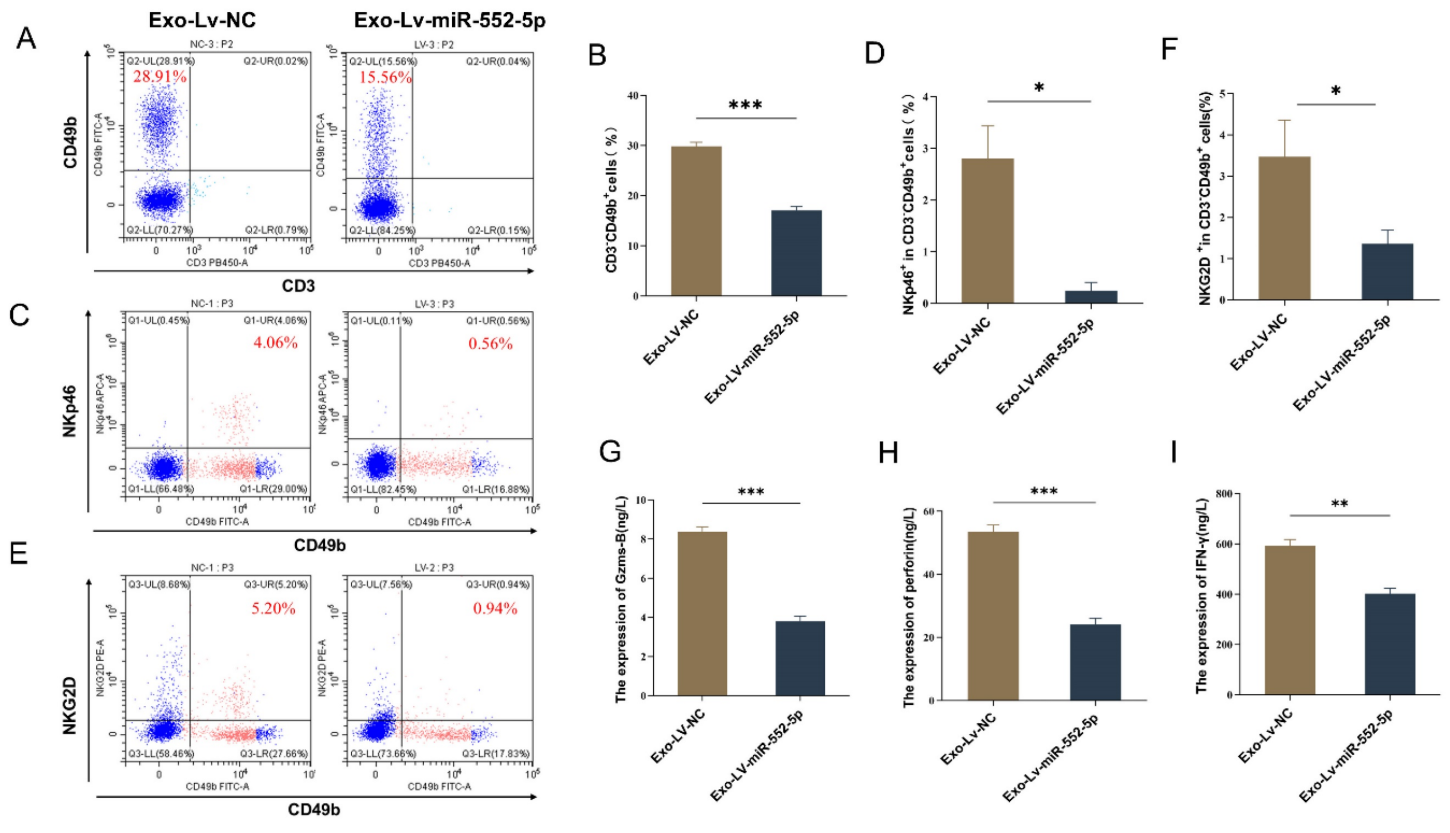
In vivo, exosomal miR-552-5p can cause NK cell failure. (A-B) NK cell percentage by flow cytometry; (C-F) NKp46 and NKG2D expression analysis by flow cytometry; (G-I) ELISA detection of the impact of exosomal miR-552-5p derived from gastric cancer cells on the secretion of Granzyme B, Perforin, and IFN-γ in NK cells from the peripheral blood of nude mice (*p < 0.05; **p < 0.01; ***p < 0.001).

To verify whether exosomal miR-552-5p could also induce NK cell dysfunction *in vivo*, AGS cells were initially transfected with a lentiviral vector containing an upregulated miR-552-5p and a negative control. This resulted in a significant upregulation of miR-552-5p expression, which was verified by qRT-PCR (Supplementary Figure 1). Subsequently, the aforementioned AGS cells were injected subcutaneously into BALB/c nude mice. After 18 days, the mice were euthanized, and peripheral blood was collected via ocular venous puncture to assess the functional status of NK cells. Due to the variability in NK cell markers among species and mouse strains, and the allelic variation of Nkrp1b and Nkrp1c in BALB/c mice, we used CD49b, NKp46, and NKG2D to identify NK cells and to test their functionality 19,20. Using flow cytometry, we first determined the percentage of NK cells in the peripheral blood of the naked mice as well as the expression of their activation receptors, NKG2D and NKp46. The proportion of NK cells and the expression of their activation receptors, NKp46 and NKG2D, in the peripheral blood of mice in the Exo-Lv-miR-552-5p group were considerably downregulated in comparison to the control group, according to the data (*p* < 0.05) (Figure 2A-F). Then, we collected the peripheral blood of the nude mice, centrifuged it to obtain serum, and used ELISA to detect the expression of cytokines Granzyme B, Perforin, and IFN-γ in the serum of each group. Results revealed that the Exo-Lv-miR-552-5p group's peripheral blood had significantly (*p* < 0.01) lower levels of Granzyme B, Perforin, and IFN-γ expression than the Exo-Lv-NC group (Figure 2G-I). This suggests that an environment where exosomal miR-552-5p is overexpressed inhibits the ability of NK cells to secrete cytokines. In summary, these results clearly indicate that exosomal miR-552-5p can induce NK cell dysfunction *in vivo*.

### Exosomal miR-552-5p inducing NK cell dysfunction via the PD-1/PD-L1 axis *in vivo*

**Figure 3:**
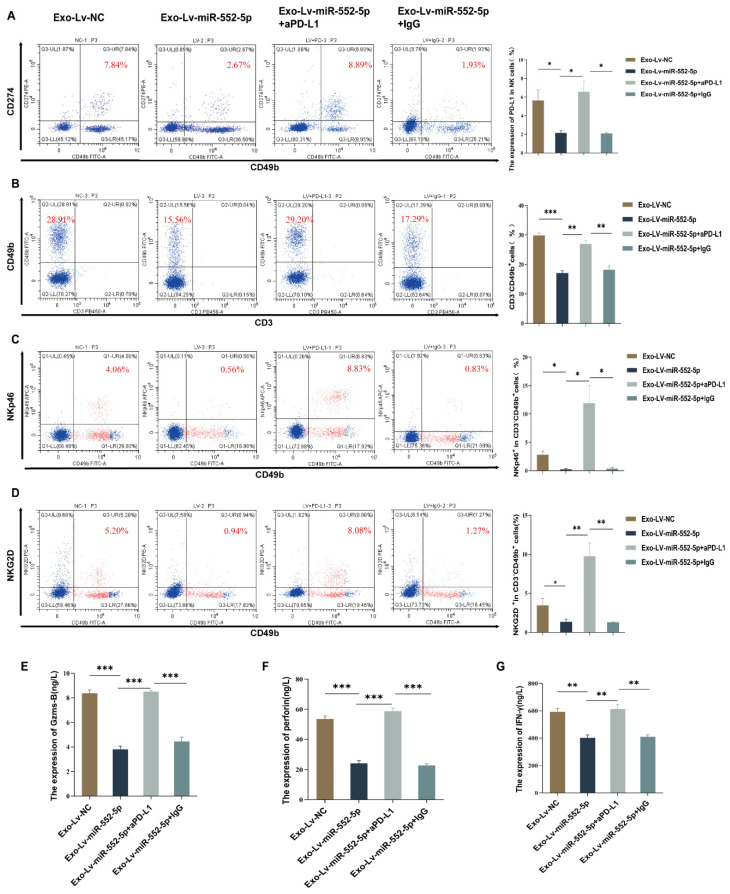
NK cell failure is induced by exosomal miR-552-5p via the PD-1/PD-L1 axis. (A) Flow cytometry analysis of PD-L1 expression levels on the surface of NK cells in the peripheral blood of nude mice from each group. (B-D) Exosomal miR-552-5p inhibits aPD-L1-induced reduction of NK cell proportions and activation receptors NKp46 and NKG2D. (E-G) Inhibition of aPD-L1 on the downregulation of Granzyme B, Perforin, and IFN-γ production in NK cells from nude mice's peripheral blood caused by exosomal miR-552-5p generated from gastric cancer cells (*p < 0.05; **p < 0.01; ***p < 0.001).

To further verify the hypothesis that exosomal miR-552-5p may cause malfunction in natural killer cells via the PD-1/PD-L1 pathway *in vivo*, we first used flow cytometry to measure the level of PD-L1 on NK cells in the peripheral blood of nude mice. The results showed that, in contrast to the Exo-Lv-NC group, the level of PD-L1 on peripheral blood NK cells was downregulated in the Exo-Lv-miR-552-5p group (*p*<0.05) (Figure 3A). Subsequently, we administered anti-PD-L1 antibodies (aPD-L1) or isotype control IgG into the peritoneal cavity of Exo-Lv-miR-552-5p group mice, and then measured the proportion of NK cells and the expression of their activation receptors NKp46 and NKG2D by flow cytometry. Additionally, we evaluated PD-L1 expression on NK cells and used ELISA to find the expression of the cytokines Granzyme B, Perforin, and IFN-γ. The results demonstrated that treatment with PD-L1 antibodies led to a considerable increase in the expression level of PD-L1 on NK cells in comparison to the group treated with IgG antibodies (*p*<0.05) (Figure 3A), the proportion of NK cells and their activation receptor expressions markedly increased (*p*<0.05) (Figure 3B-D), and the cytokines Granzyme B, Perforin, and IFN-γ all had their levels of expression restored (*p*<0.01) (Figure 3E-G). These results imply that exosomal miR-552-5p can cause NK cell malfunction via the PD-1/PD-L1 axis *in vivo*.

### Exosomal miR-552-5p regulates NK Cells to promote EMT of gastric cancer cell *in vitro*

**Figure 4:**
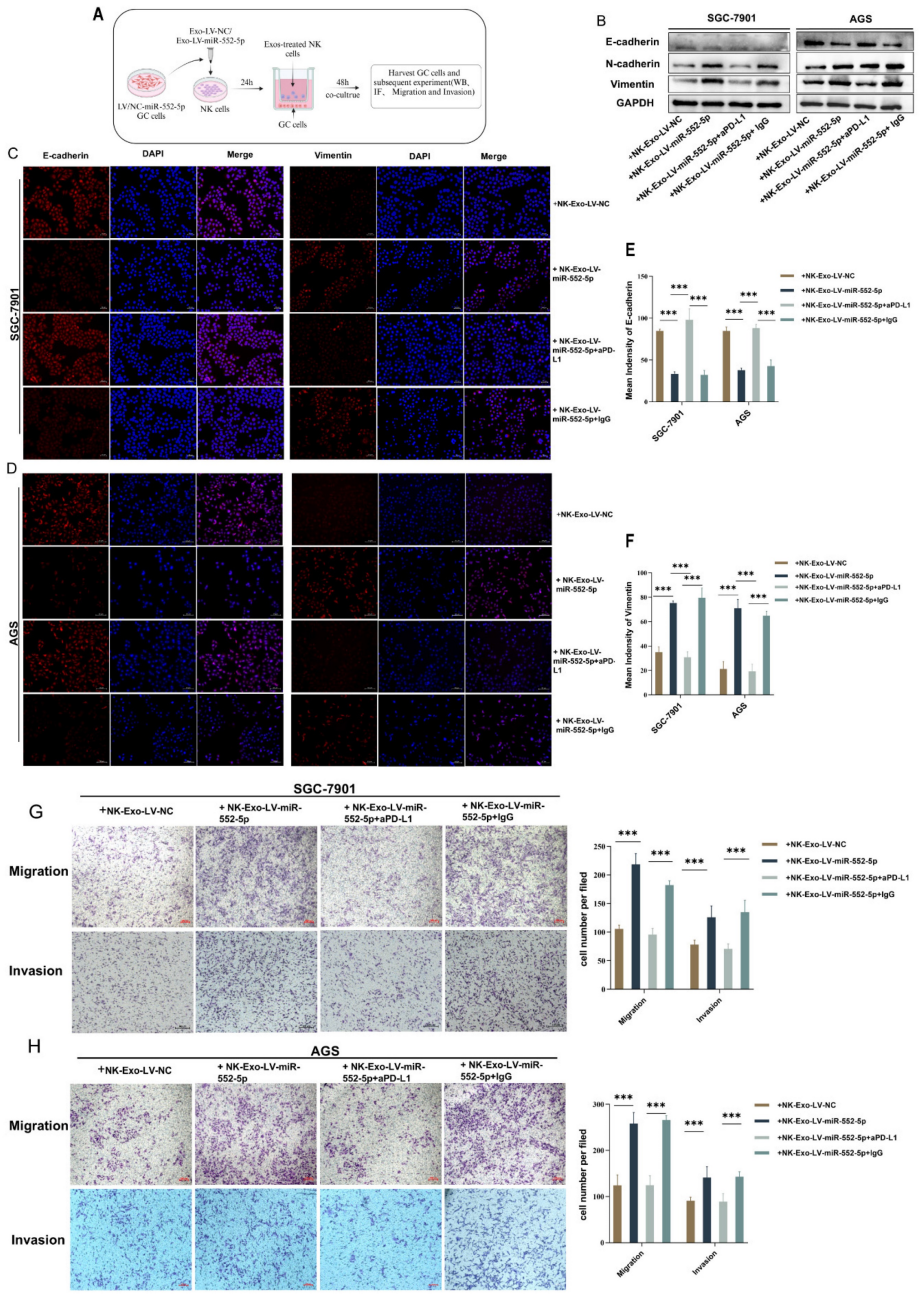
*In vitro* verification of exosomal miR-552-5p regulating NK cells to promote the EMT process of GC cells. (A) Diagram representing the *in vitro* co-culture model of GC cells and NK cells that have been pretreated with exosomal miR-552-5p. (B-F) Western blotting and IF examination of the EMT-related protein expression levels in each group's GC cells. (G-H) Transwell and invasion assays were used to assess the migration and invasion potential of each group's GC cells (*p < 0.05; **p < 0.01; ***p < 0.001).

Online analysis using the TIMER 2.0 website revealed a positive correlation between the expression of E-cadherin protein in GC cells and the NK cells (Supplementary Figure 2), suggesting that the deficiency or inactivation of NK cells may promote gastric cancer EMT. To further explore the impact of NK cells pretreated with exosomal miR-552-5p on the EMT of GC cells, we established a co-culture system of NK cells and gastric cancer cells. Initially, equal volumes of sterile exosomes, Exo-Lv-miR-552-5p or Exo-Lv-NC, were added to the NK-92 cell culture medium and co-cultured for 24 hours to pretreat NK cells. The pretreatment NK cells were then co-cultured with GC cells SGC-7901/AGS for 48 hours using a non-contact co-culture system (Figure 4A). Western blotting and IF assays were used to look for changes in the EMT markers of GC cells. The outcomes demonstrated that the protein levels of E-cadherin in GC cells dropped while the expression of mesenchymal markers N-cadherin and vimentin rose following co-culturing with NK cells prepped with Exo-Lv-miR-552-5p for 48 hours (Figure 4B), and similar phenomena were observed in the IF assays (*p*<0.001) (Figure 4C-F). Using the transwell migration and invasion tests, we further examined the impact of NK cells pretreatment with exosomal miR-552-5p on the migratory and invasion capacities of GC cells. The findings demonstrated that following co-culturing for 48 hours with NK cells prepped with Exo-Lv-miR-552-5p, the migratory and invasion capacities of the SGC-7901 and AGS cell lines were improved in comparison to the control(*p*<0.001) (Figure G-H). Furthermore, when anti-PD-L1 antibodies (0.05mg/mL) or their isotype control antibodies IgG were added to the culture medium where NK cells were co-cultured with Exo-Lv-miR-552-5p, and the EMT status of GC cells was verified, and the results showed opposite changes after treatment with anti-PD-L1 antibodies (*p*<0.001). According to the study's findings, exosomal miR-552-5p can cause NK cell malfunction via the PD-1/PD-L1 axis, hence increasing GC cell EMT, migration, and invasion *in vitro*.

### Validation of exosomal miR-552-5p regulating NK cells to promote the growth and EMT of gastric cancer cells *in vivo*

**Figure 5:**
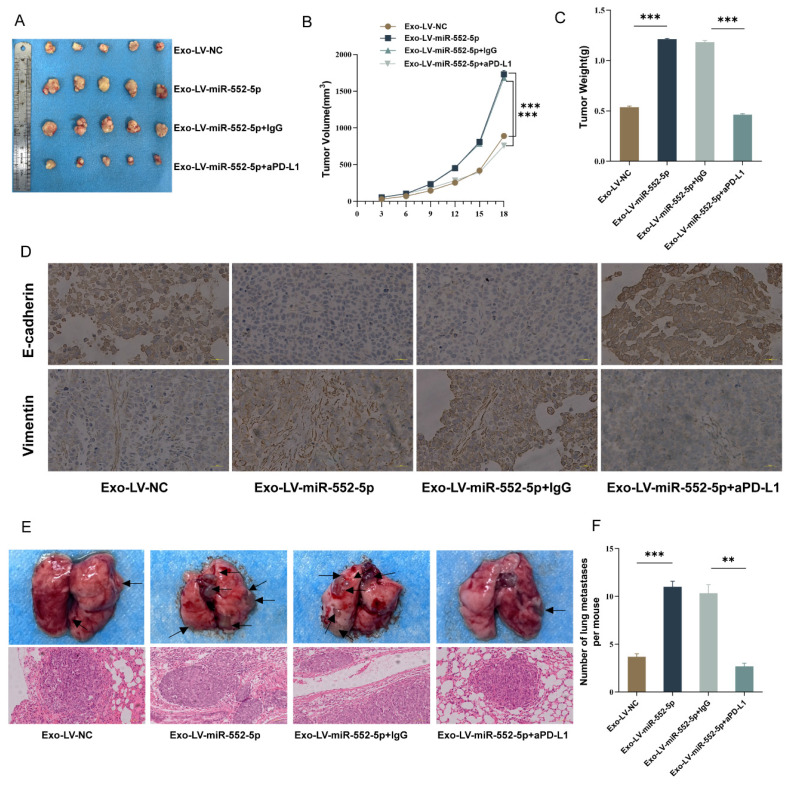
In vivo outcomes indicate exosomal miR-552-5p influences NK cell function and promotes GC cell development and EMT via the PD-1/PD-L1 axis. (A) Images of subcutaneously implanted tumors in nude mice from each group. (B) Growth curves showing each group's implanted tumors in nude mice. (C) The tumor weight of the transplanted tumors in the group of nude mice. (D) IHC shows the expression levels of Vimentin and E-cadherin (scale bar, 20 μm). (E) H&E staining to identify representative photographs of lung metastatic nodules in nude mice and representative pictures of lung metastasis in each group. (F) The quantity of lung metastatic nodules in each group's nude mice (*p < 0.05; **p < 0.01; ***p < 0.001).

We used tail vein injection techniques and subcutaneous tumor growth in nude mice to confirm the possible function of exosomal miR-552-5p in NK cells and gastric cancer EMT *in vivo*. The subcutaneously implanted tumors in the Exo-Lv-miR-552-5p treatment group were much larger than those in the Exo-Lv-NC treatment group, according to the results of the subcutaneous tumor formation experiment (*p*<0.001) (Figure 5A). Exo-Lv-miR-552-5p treatment group subcutaneously implanted tumors showed a significant rise in both volume (Figure 5B) and weight (Figure 5C) according to quantitative analysis (*p*<0.001). Vimentin expression was dramatically elevated in the tumor cells of the subcutaneous tumor tissue in the Exo-Lv-miR-552-5p group, whereas E-cadherin expression decreased significantly, according to IHC analysis (Figure 5D). In the nude mice tail vein injection tumor development experiment, it was found that the Exo-Lv-miR-552-5p treatment group increased AGS cell distant metastasis than the control group (*p*<0.05). Moreover, these effects were reversed after treatment with anti-PD-L1 antibodies (all* p*<0.001) (Figure 5E-F). The outcomes of NK cells in the peripheral blood of the mice in each group, along with the *in vivo* experimental results that matched the *in vitro* studies, verified that exosomal miR-552-5p derived from GC influences NK cell function via the PD-1/PD-L1 axis, thereby promoting the growth of subcutaneous transplanted tumors, EMT, and distant metastasis of GC cells in nude mice.

## Discussion

In the realm of oncology, miRNAs have emerged as pivotal biomarkers for gauging tumor progression, with their stability enhanced when encapsulated within exosomes in plasma 21. Current studies show that exosome-encased circulating miRNAs can be transported across cells, affecting immunotherapy, tumor cell invasion, and the tumor microenvironment 12,22. On chromosome 1p34.3, a short non-coding RNA called miR-552 is overexpressed in a number of malignancies, including stomach cancer 23. Our study delves into the intricate relationship between exosomal miR-552-5p, the PD-1/PD-L1 axis, and NK cell function, providing novel insights into the regulatory mechanisms that govern GC progression and EMT. While previous research, including our own, has established the role of exosomal miR-552-5p in tumorigenesis and disease progression via the PTEN/TOB1 axis 13, the current work expands upon these findings by elucidating a distinct pathway through which exosomal miR-552-5p may modulate immune responses.

A critical component of the immune system, NK cells are known to produce a plethora of chemokines and immune regulatory cytokines, including IFN-γ and TNF-α. They also release perforin and granzyme B, which directly enhance cytotoxicity and limit tumor spread 6,7.

The activity and function of NK cells have been tightly linked to exosomal miRNAs in many studies on neuroblastoma, breast cancer, and ovarian cancer 24,25. In our recent work, we found that exosomal miR-552-5p levels inversely track with NK cell phenotypic distribution and activated receptor expression in gastric cancer patients^14^. Evidence suggests that exosomes can modulate NK cell function by inhibiting receptor and cytokine production, potentially impairing their anti-tumor capabilities and further suppressing the immune response 26-28. Our study builds upon this foundation by providing evidence that exosomal miR-552-5p significantly diminishes NK cell activity in vivo, potentially facilitating tumor progression.

Studies have shown that NK cells can influence the growth of tumors by upregulating the expression of PD-L1 in tumor cells 29. Studies conducted recently have demonstrated that PD-L1 is highly expressed on specific immune cells, including NK cells, which are in addition to the surface of tumor cells. These NK cells that express PD-L1 are also more effective in destroying tumors 30-32. Our findings show that exosomal miR-552-5p can lower PD-L1 expression on the surface of NK cells, reducing their immunological impact. The pivotal role of the PD-1/PD-L1 axis in the modulatory effect of exosomal miR-552-5p on NK cells is underscored by the restoration of NK cell-activated receptor expression, upregulation of PD-L1 on NK cells, and enhanced cytokine secretion observed in nude mice following treatment with anti-PD-L1 antibodies.

**Figure 6:**
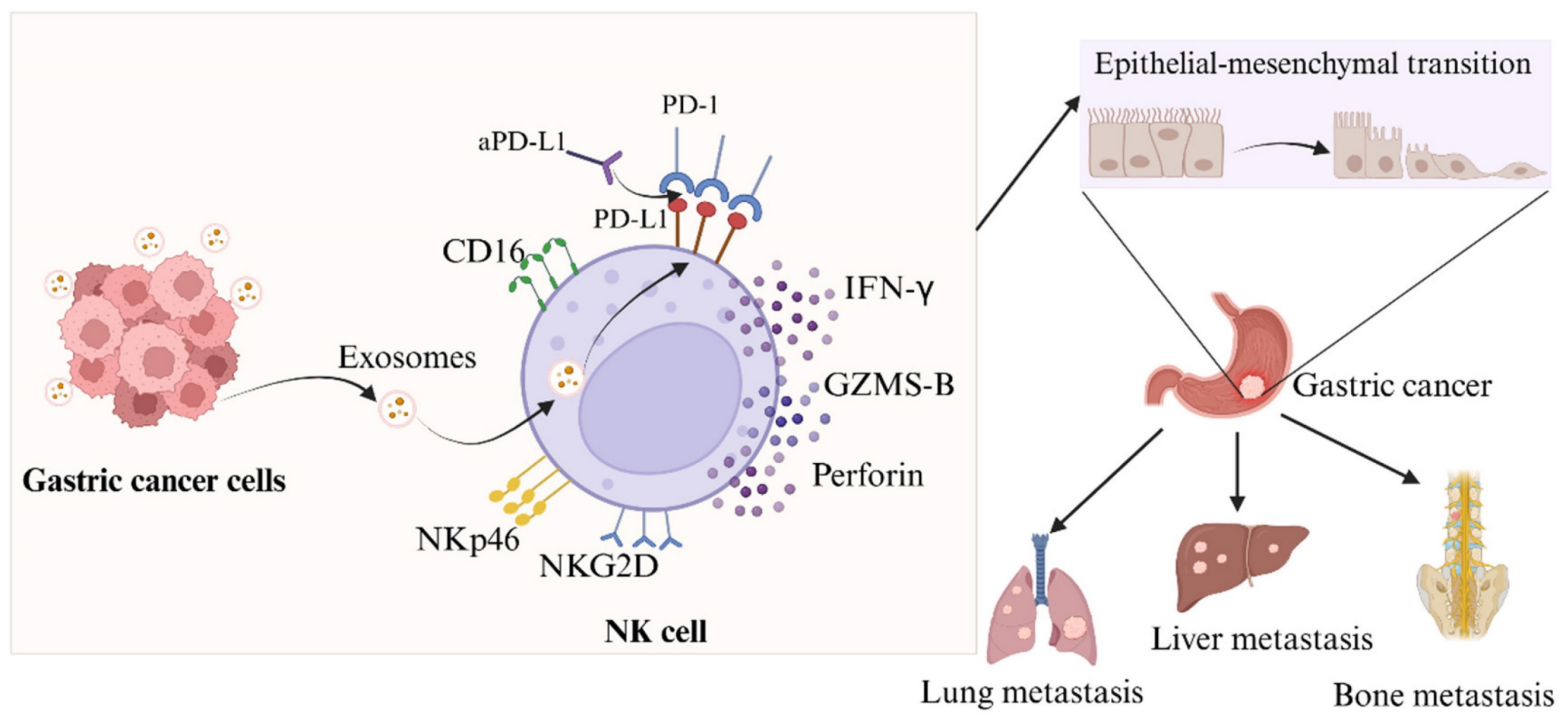
Diagram illustrating the way in which exosomal miR-552-5p controls NK cells in EMT of gastric cancer via the PD-1/PD-L1 axis (created by Biorender.com).

When it comes to the occurrence and progression of cancer metastasis, EMT is a crucial stage. While N-cadherin and vimentin expression is linked to enhanced EMT and metastasis, e-cadherin can effectively limit the invasion and development of epithelial malignancies 33,34. Evidence suggests that compromised NK cell function or reduced activity can drive tumor EMT via various pathways 35,36, with patient outcomes linked to the extent of NK cell infiltration 37. We have identified that NK cells were rendered immunosuppressive by exosomal miR-552-5p. We proceeded to elucidate the relationship between the EMT in GC cells and NK cells following the intervention of exosomal miR-552-5p. According to our findings, as illustrated in Figure 6, exosomal miR-552-5p could facilitate EMT, invasion, migration and metastasis of GC cells by impairing NK cell function.

## Conclusion

In summary, our results validate the notion that exosomal miR-552-5p stimulates immunological escape and promotes gastric cancer EMT, with the PD-1/PD-L1 Axis leading to NK cell immunosuppression. These findings offer a novel perspective and a potential therapeutic strategy for GC immunotherapy. Further investigation is warranted to determine if exosomal miR-552-5p influences PD-L1 expression on tumor cells while controlling NK cell immunological activity.

## Supplementary Material

Supplementary figures.
