# Supplementary material for: Exosomal miR-552-5p Regulates the Role of NK Cells in EMT of Gastric Cancer via the PD-1/PD-L1 Axis
Source: J Cancer. 2025 Jan 1;16(2):406–16. doi: 10.7150/jca.102360 (PMC11685699; doi:10.7150/jca.102360)
Supplement: Supplementary file 1 — Supplementary figures. [file jcav16p0406s1.pdf]

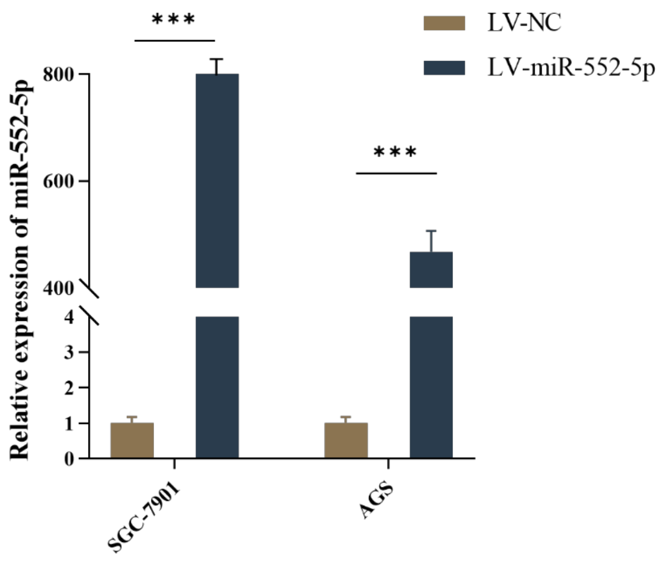

Supplementary figure 1. A qRT-PCR analysis of miR-552-5p expression in lentiviral vector-transfected cells for overexpression of miR-552-5p and the correlating.

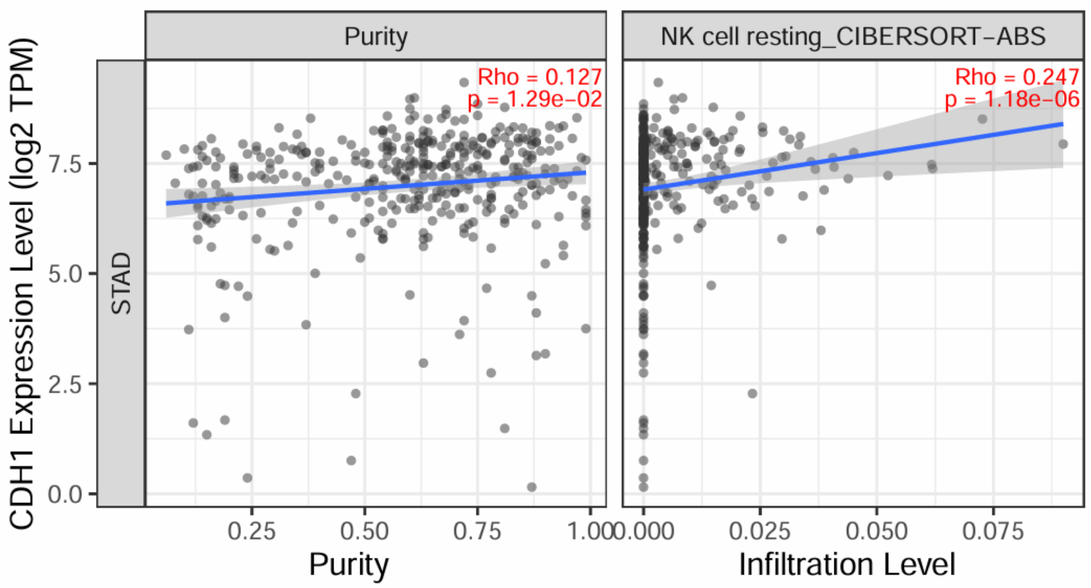

Supplementary figure 2. Positive correlation between the expression levels of E-cadherin protein and the infiltration degree of NK cells in gastric cancer.
